# Factors associated with selection of total hip arthroplasty versus hemiarthroplasty for femoral neck fracture in older adults: a nationwide analysis of 99,084 cases

**DOI:** 10.1007/s00402-026-06459-1

**Published:** 2026-08-10

**Authors:** David Maman, Yaniv Steinfield, Yaron Berkovich

**Affiliations:** 1 Carmel Medical Center, Haifa, Israel; 2 The Ruth and Bruce Rappaport Faculty of Medicine, Technion – Israel Institute of Technology, Haifa, Israel

**Keywords:** Femoral neck fracture, Total hip arthroplasty, Hemiarthroplasty, Patient selection

## Abstract

**Background:**

Surgical management of displaced femoral neck fractures in older adults typically involves hemiarthroplasty or total hip arthroplasty (THA). Although clinical guidelines suggest that THA may be considered in selected healthier and cognitively intact patients, real-world procedure selection varies widely. This study evaluated patient factors associated with selection of THA versus hemiarthroplasty in a contemporary U.S. dataset.

**Methods:**

A retrospective cohort study was conducted using the 2022 Nationwide Readmissions Database (NRD). Patients ≥ 65 years hospitalized with femoral neck fracture were identified using ICD-10-CM codes. Those treated with internal fixation or non-arthroplasty procedures were excluded. Weighted analyses characterized demographics, comorbidities, and hospital utilization between THA and hemiarthroplasty groups. A multivariable logistic regression model identified factors independently associated with receiving THA. All analyses accounted for NRD survey design.

**Results:**

Among 142,013 operative cases, 99,084 met inclusion criteria (81.8% hemiarthroplasty; 18.2% THA). Patients receiving THA were younger (76.6 vs. 81.9 years), had shorter length of stay (6.06 vs. 7.24 days), and were more frequently discharged home (17.5% vs. 6.2%). Metabolic conditions were associated with increased odds of THA, including obesity (OR 1.17) and sleep apnea (OR 1.12). Frailty-related conditions were associated with markedly reduced THA likelihood, including Alzheimer’s disease (OR 0.48), Parkinson disease (OR 0.55), chronic kidney disease (OR 0.78), chronic lung disease (OR 0.72), and congestive heart failure (OR 0.77). Each additional year of age decreased the odds of THA by approximately 9%.

**Conclusion:**

In this nationwide cohort, selection of THA rather than hemiarthroplasty was associated with younger age and lower prevalence of frailty- and cognition-related comorbidities, while several metabolic comorbidities showed modest positive associations with THA use. These findings describe contemporary national selection patterns but do not establish treatment appropriateness, clinical benefit, or guideline concordance, as key factors such as pre-fracture mobility, functional independence, and living situation were not available in the dataset.

**Level of evidence:**

Level III.

## Introduction

Femoral neck fractures are among the most consequential fragility injuries in older adults, frequently resulting in substantial pain, loss of mobility, and long-term functional decline, with major downstream effects on independence and health-system utilization [1]. As populations age globally, the burden of hip fractures is expected to remain high and continue rising in many settings, amplifying clinical and economic pressures on healthcare systems [1]. 

For displaced intracapsular femoral neck fractures in older adults, arthroplasty is the dominant surgical strategy, with hemiarthroplasty and total hip arthroplasty (THA) representing the two principal options. Contemporary guidelines generally support consideration of THA in carefully selected patients who are community ambulators, cognitively intact, and medically fit, while hemiarthroplasty is typically preferred for frailer patients with limited functional reserve or substantial comorbidity [2, 3]. Despite these recommendations, multiple population-based studies have demonstrated meaningful variation in THA utilization across institutions and patient subgroups, suggesting that real-world procedure selection is influenced by factors beyond guideline criteria alone [4, 5]. 

Prior comparative literature on THA versus hemiarthroplasty has largely emphasized clinical outcomes (e.g., function, dislocation, reoperation, mortality) and resource utilization. In the landmark HEALTH randomized trial, THA provided modest functional advantages but was associated with a higher risk of certain serious adverse events, reinforcing the importance of patient selection [6]. However, comparatively fewer investigations have focused on how surgeons choose between THA and hemiarthroplasty in routine practice at a national level, and existing work has often relied on regional cohorts or administrative datasets from earlier eras [4, 5]. At the same time, utilization trends suggest that THA is increasingly used for fracture indications in selected older adults, heightening the relevance of characterizing contemporary selection patterns.

Understanding nationwide patterns of procedure selection remains important for several reasons. First, describing patient characteristics associated with THA versus hemiarthroplasty may help identify practice variation and potential disparities in use [2–5]. Second, such data may clarify how measurable administrative variables relate to real-world surgical selection at a national level. However, because administrative datasets do not capture key clinical factors such as pre-fracture ambulatory status, functional independence, living situation, and formal cognitive assessment, these analyses should be interpreted as descriptive of observed selection patterns rather than evidence of optimal treatment selection or guideline-concordant care.

Using the 2022 Nationwide Readmissions Database (NRD) a large, nationally representative all-payer U.S. inpatient dataset designed for weighted analyses we sought to [1] describe the distribution of operative procedures performed for femoral neck fracture in older adults; [2] compare demographic, clinical, and hospital characteristics between patients undergoing THA and those receiving hemiarthroplasty; and [3] identify independent predictors of receiving THA rather than hemiarthroplasty using a survey-weighted multivariable logistic regression model [7]. By analyzing 99,084 arthroplasty cases, this study provides a contemporary national assessment of patient-level factors associated with arthroplasty selection for femoral neck fracture.

## Materials and methods

### Data source

This retrospective cohort study used data from the 2022 Nationwide Readmissions Database (NRD), part of the Healthcare Cost and Utilization Project (HCUP) of the Agency for Healthcare Research and Quality. The NRD contains all-payer inpatient discharge records from 30 participating U.S. states and is designed to allow nationally weighted estimation of hospitalizations and readmissions. Each record includes patient demographics, diagnoses and procedures coded using ICD-10-CM/PCS, hospital characteristics, discharge disposition, charges, length of stay, and a unique visit linkage number.

### Study population

We identified all patients aged 65 years or older who were hospitalized with femoral neck fracture in 2022. Femoral neck fractures were defined using ICD-10-CM diagnosis codes for intracapsular proximal femur fractures (S72.00-S72.09). Patients younger than 65 years and those with missing key demographic or procedural data were excluded.

### Surgical procedure categorization

Patients were grouped according to the primary procedure performed during the index hospitalization:

- Internal fixation (cannulated screws, dynamic hip screw, cephalomedullary nail).
- Hemiarthroplasty.
- Total hip arthroplasty (THA).
- Other procedure or no procedure.

Procedure categories were defined using ICD-10-PCS codes. Because the study objective was to compare hemiarthroplasty with THA, patients undergoing internal fixation or other procedures were excluded from the final analytic cohort.

#### Primary outcome

The primary outcome was the type of arthroplasty performed: total hip arthroplasty (THA) vs. hemiarthroplasty.

#### Secondary outcomes

Secondary clinical and utilization outcomes compared descriptively between groups included:

- In-hospital mortality.
- Length of stay (days).
- Total hospital charges (USD).

Discharge disposition (home, home health care, skilled nursing facility/rehabilitation/long-term care, short-term hospital transfer, against medical advice, in-hospital death).

These outcomes were analyzed for descriptive context but were not included as predictors in the multivariable model to avoid introducing post-exposure variables.

### Covariates

Demographic covariates included age (continuous) and sex.

Clinical covariates included comorbidities identified using ICD-10-CM codes.

Hypertension, dyslipidemia, sleep apnea, chronic anemia, smoking, alcohol abuse, osteoporosis, mental disorders, Parkinson disease, Alzheimer’s disease, chronic kidney disease, congestive heart failure (CHF), chronic lung disease, thyroid disorders, liver disease, diabetes mellitus, history of myocardial infarction (MI), peripheral vascular disease, history of cerebrovascular accident (CVA), dementia, peptic ulcer disease, hemiplegia, neoplasms, and obesity.

The NRD does not provide a direct body mass index (BMI) variable; therefore, obesity was captured using diagnosis codes. Race/ethnicity variables are available in the NRD; however, these were not included in the primary analysis in order to prioritize clinical comorbidities and frailty-related factors most directly relevant to surgical decision-making and to maintain model parsimony. Inclusion of race/ethnicity may provide additional insight into disparities in procedure selection and represent an important area for future investigation.

### Statistical analysis

Continuous variables were summarized as means with standard deviations and compared using independent-samples t-tests. Categorical variables were summarized as frequencies with percentages and compared using χ² tests. All analyses accounted for NRD sampling weights, strata, and clusters according to HCUP recommendations to produce nationally representative estimates.

A multivariable logistic regression model was constructed to identify independent predictors of receiving THA rather than hemiarthroplasty. The dependent variable was (1 = THA, 0 = hemiarthroplasty). All demographic and clinical covariates were entered simultaneously. Odds ratios (ORs) with 95% confidence intervals (CIs) and p-values were reported.

Model diagnostics included:

- Hosmer-Lemeshow goodness-of-fit test.
- Examination of variance inflation factors for multicollinearity.
- Evaluation of model convergence and stability of estimates.

A two-sided p-value < 0.05 was considered statistically significant. Statistical analyses were performed using IBM SPSS.

### Missing data

Variables with missingness < 1% were analyzed using complete-case methods. No variable exceeded HCUP thresholds requiring imputation, and patterns of missing data were determined to be random at the dataset level.

### Ethical considerations

The NRD is a publicly available, fully deidentified dataset; therefore, the study did not require institutional review board approval or informed consent according to federal guidelines.

## Results

In the 2022 Nationwide Readmissions Database, 142,012 patients aged 65 years or older underwent operative treatment for femoral neck fracture. Hemiarthroplasty was the most common procedure, performed in 81,003 patients (57.0%), followed by internal fixation in 30,483 patients (21.5%), total hip arthroplasty (THA) in 18,081 patients (12.7%), and other or unspecified procedures in 12,445 patients (8.8%) (Table 1).

**Table 1:** Distribution of surgical procedure types in NRD 2022

Procedure type	Frequency	Percent
Internal Fixation	30,483	21.5
Hemiarthroplasty	81,003	57.0
Total Hip Arthroplasty	18,081	12.7
Other procedure/No procedure	12,445	8.8
Total	142,012	100.0

Because the objective of this study was to compare outcomes between hemiarthroplasty and THA, patients treated with internal fixation or other procedures were excluded. The final analytic cohort consisted of 99,084 arthroplasty patients, of whom 81,003 (81.8%) underwent hemiarthroplasty and 18,081 (18.2%) underwent THA (Table 2).

**Table 2:** Analytic cohort: hemiarthroplasty vs. total hip arthroplasty

Procedure type	Frequency	Percent
Hemiarthroplasty	81,003	81.8
Total Hip Arthroplasty	18,081	18.2
Total	99,084	100.0

Baseline characteristics and hospitalization metrics for the analytic cohort are presented in Table 3. Patients undergoing total hip arthroplasty were younger (76.6 vs. 81.9 years) and slightly less often female (65.6% vs. 67.6%). Medicare was the primary payer for 89.4% of THA patients and 93.2% of hemiarthroplasty patients. Length of stay was shorter after THA (6.06 vs. 7.24 days), while total hospital charges were slightly higher (108,847 USD vs. 103,617 USD). In-hospital mortality remained low overall but differed substantially between groups, occurring in 0.7% of THA cases compared with 1.9% among hemiarthroplasty patients.

**Table 3:** Demographics and hospital utilization outcomes by procedure type

Variable	Hemiarthroplasty (*n* = 81,003)	Total Hip Arthroplasty (*n* = 18,081)	*p*-value
Age, mean ± SD (years)	81.93 ± 6.97	76.55 ± 7.27	< 0.001
Female (%)	67.6	65.6	< 0.001
Primary payer: Medicare (%)	93.2	89.4	< 0.001
Length of stay, mean ± SD (days)	7.24 ± 6.21	6.06 ± 5.54	< 0.001
Total hospital charges, mean ± SD (USD)	103,617 ± 78,141	108,847 ± 79,055	< 0.001
In-hospital mortality (%)	1.9	0.7	< 0.001

Discharge disposition differed substantially between groups and is presented in Table 4. Patients undergoing hemiarthroplasty were most commonly discharged to skilled nursing facilities, rehabilitation centers, or long-term care (73.6%), whereas this occurred in only 49.1% of total hip arthroplasty patients. Conversely, discharge home occurred nearly three times more frequently after total hip arthroplasty (17.5%) compared with hemiarthroplasty (6.2%). Use of home health services was also higher in the total hip arthroplasty group (32.2% vs. 17.7%). In-hospital mortality, consistent with earlier results, was significantly more common among hemiarthroplasty patients (1.9% vs. 0.7%). These differences were statistically significant (*p* < 0.001).

**Table 4:** Discharge disposition by procedure type

Disposition category	Hemiarthroplasty (%)	Total Hip Arthroplasty (%)
Home	6.2	17.5
Short-term hospital transfer	0.5	0.4
SNF/Rehab/Long-term care	73.6	49.1
Home health care	17.7	32.2
Against medical advice	0.1	0.1
Died in hospital	1.9	0.7

**Fig. 1:**
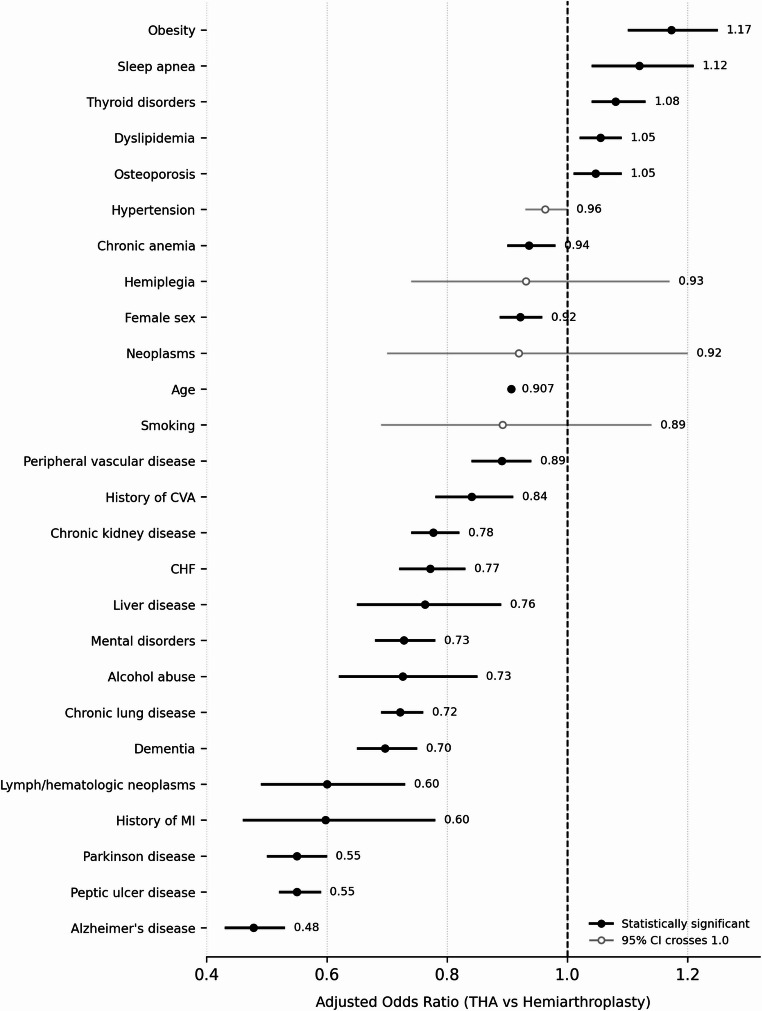
Predictors of receiving total hip arthroplasty vs. hemiarthroplasty Adjusted odds ratios for factors associated with receiving total hip arthroplasty rather than hemiarthroplasty. The vertical dashed line represents OR = 1.0. Confidence intervals crossing 1.0 indicate non-statistically significant associations

In the multivariable logistic regression model evaluating factors associated with receiving total hip arthroplasty (THA) rather than hemiarthroplasty, several patient characteristics demonstrated significant associations (Fig. 1).

Obesity (OR 1.17, 95% CI 1.10–1.25, *p* < 0.001), sleep apnea (OR 1.12, 95% CI 1.04–1.21, *p* = 0.004), thyroid disorders (OR 1.08, 95% CI 1.04–1.13, *p* < 0.001), and dyslipidemia (OR 1.06, 95% CI 1.02–1.09, *p* = 0.003) were associated with increased odds of receiving THA. These findings indicate that patients with metabolic or endocrine comorbidities were modestly more likely to undergo THA.

By contrast, a wide range of clinical conditions were associated with significantly lower likelihood of receiving THA. These included Alzheimer’s disease (OR 0.48, 95% CI 0.43–0.53), peptic ulcer disease (OR 0.55, 95% CI 0.52–0.59), Parkinson disease (OR 0.55, 95% CI 0.50–0.60), history of myocardial infarction (OR 0.60, 95% CI 0.46–0.78), and chronic kidney disease (OR 0.78, 95% CI 0.74–0.82), all with *p* < 0.001. Several additional comorbidities showed similar associations, including congestive heart failure, chronic lung disease, liver disease, dementia, alcohol abuse, and mental disorders, with odds ratios generally ranging from 0.69 to 0.77 and all *p* < 0.001. This pattern reflects a broad tendency to avoid THA in patients with systemic frailty or cognitive impairment.

Age demonstrated a strong independent effect, with each additional year lowering the odds of receiving THA by approximately 9% (OR 0.91, 95% CI 0.905–0.909, *p* < 0.001). Female sex was also associated with lower use of THA (OR 0.92, 95% CI 0.887–0.958, *p* < 0.001). Assessment of multicollinearity demonstrated no evidence of problematic collinearity among model covariates, with all variance inflation factors below 2.0.

Overall, THA use was associated with patient age, frailty-related comorbidities, chronic cardiopulmonary disease, cognitive disorders, and selected metabolic profiles. Figure 1 presents the complete set of adjusted odds ratios and confidence intervals for all predictors.

## Discussion

In this nationally representative cohort of 99,084 older adults undergoing arthroplasty for femoral neck fracture, several important findings emerged regarding real-world patterns of procedure selection. First, THA accounted for 18.2% of arthroplasty procedures, consistent with prior reports demonstrating increasing utilization of THA for fracture indications over the past decade [8, 9]. Second, substantial differences were observed in baseline characteristics between patients receiving THA and those undergoing hemiarthroplasty, highlighting the association between patient-level factors and procedure selection [10, 11]. Third, multivariable analysis revealed a consistent pattern in which metabolic and endocrine comorbidities were associated with slightly increased odds of THA, whereas frailty-associated, cognitive, and systemic disease factors were strongly associated with decreased THA utilization [6, 12].

Conditions such as Alzheimer’s disease, dementia, Parkinson disease, chronic kidney disease, chronic lung disease, congestive heart failure, and liver disease were all associated with significantly lower odds of THA. These associations are directionally consistent with the broader clinical literature suggesting that THA is more often used in healthier and less frail patients; however, our data cannot determine whether these observed selection patterns were appropriate, beneficial, or guideline-concordant for individual patients because key factors such as pre-fracture mobility, independence, living situation, and formal cognitive assessment are not captured in the NRD [13–15]. The magnitude of the observed associations highlights the strong relationship between measurable frailty-related comorbidities and real-world procedural selection in administrative data [12, 16]. 

Age demonstrated a particularly pronounced effect, with each additional year associated with an approximately 9% reduction in the likelihood of receiving THA. Similar age-dependent patterns have been reported in prior national and regional analyses and likely reflect the combined influence of increasing frailty, comorbidity burden, and reduced postoperative functional expectations among the oldest patients [8, 10, 17]. Although age alone is an imperfect surrogate for functional status, it remains a central consideration in arthroplasty decision-making for hip fracture patients [13, 15].

In contrast, obesity, dyslipidemia, sleep apnea, and thyroid disorders were associated with modest increases in THA utilization. These conditions may serve as markers of a distinct health phenotype characterized by preserved ambulation and community dwelling despite chronic disease, rather than classic frailty [11, 18]. Alternatively, these findings may reflect residual confounding related to unmeasured functional status or social support factors that influence surgeon perception of a patient’s capacity to benefit from THA [12, 16]. Importantly, effect sizes were small, and these associations should be interpreted as correlates of selection rather than causal drivers of procedure choice [6, 17].

Female sex was associated with slightly lower THA utilization, consistent with prior population-based studies [10, 11]. Potential explanations include sex-related differences in bone density, baseline mobility, living arrangements, or surgeon preference, although unmeasured functional variables likely contribute to this association [17].

Marked differences in discharge disposition further support the role of baseline functional status in procedure selection. Patients undergoing THA were substantially more likely to be discharged home or with home health services, whereas those treated with hemiarthroplasty more frequently required skilled nursing or long-term care [9, 12]. Although discharge disposition represents a post-treatment variable and cannot be interpreted as a determinant of procedure choice, these findings are consistent with prior literature linking THA selection to higher pre fracture independence and anticipated rehabilitation potential [13, 14, 16].

Previous comparative studies of THA versus hemiarthroplasty have largely focused on postoperative outcomes, demonstrating modest functional advantages of THA in selected patients alongside higher risks of instability and revision [15, 17]. The present study adds to this literature by focusing on contemporary nationwide selection patterns rather than postoperative outcomes, providing a large-scale description of which measurable demographic and comorbidity variables were associated with THA versus hemiarthroplasty use [8, 10, 12]. These findings should not be interpreted as evidence that surgeons selected the optimal procedure or that patients benefited from this selection strategy, but rather as a description of how arthroplasty choice was associated with the limited variables available in an administrative dataset.

From a clinical perspective, these results primarily identify measurable administrative variables associated with arthroplasty selection in routine practice. They may help highlight practice variation and generate hypotheses regarding how patient comorbidity profiles relate to procedural choice across institutions [10, 11]. However, they should not be interpreted as validating a selection strategy, demonstrating patient benefit, or establishing best practice, because outcome validation and key pre-fracture functional variables were unavailable in the present dataset [12, 16, 18, 19]. 

Strengths of this study include the large, nationally representative cohort, standardized coding, and rigorous survey-weighted analytic approach [6]. Limitations include the use of administrative data, which lack granular clinical details such as fracture displacement, pre-fracture ambulatory capacity, functional independence, living situation, formal cognitive assessment, and radiographic findings [12, 17]. In addition, procedural details such as surgical approach, fixation method (cemented vs. uncemented), implant design including dual-mobility constructs, and femoral head size are not available in the NRD and could not be evaluated. Hospital- and surgeon-level factors, including hospital volume, teaching status, institutional protocols, and surgeon subspecialty, were not explicitly modeled and may also contribute to variation in procedure selection. Social determinants and support systems, including caregiver availability, socioeconomic context, and regional resource availability, are not captured in the NRD and may influence both procedure selection and discharge disposition. Because this study is based on a single-year cross-sectional dataset, temporal trends in THA utilization and changes in institutional practice patterns over time could not be assessed. In addition, this study did not evaluate postoperative functional outcomes, quality of life, complications, reoperations, dislocation, or long-term survival in relation to the observed selection factors. Accordingly, the findings should be interpreted as descriptive associations with procedure selection rather than evidence of appropriateness, clinical usefulness, or optimal care. Residual confounding is therefore possible, and causal inferences cannot be drawn. Future studies using longitudinal multi-year data would help clarify temporal trends in THA utilization and evolving selection patterns. Nevertheless, the consistency and magnitude of observed associations across multiple frailty and cognitive domains support the clinical plausibility and relevance of the findings [13–15]. Given the increasing use of THA for fracture indications reported in prior literature, future longitudinal analyses spanning multiple years would be particularly valuable to better characterize evolving utilization patterns and institutional practice variation.

## Conclusion

Selection of THA versus hemiarthroplasty for femoral neck fracture in older adults was associated with age, frailty-related comorbidities, cognitive disorders, and systemic disease burden, while several metabolic and endocrine conditions showed modest positive associations with THA use. These findings describe contemporary national selection patterns within the constraints of administrative data. They do not establish treatment appropriateness, guideline concordance, or patient benefit, and should be interpreted in light of the absence of key clinical variables such as pre-fracture mobility, functional independence, living situation, and formal cognitive assessment.

## Data Availability

NRD data are available from HCUP by data use agreement; ICD-10 code lists and balance diagnostics can be provided as supplementary materials on request.
